# Ratio of positive lymph nodes: The prognostic value in stage IV thyroid cancer

**DOI:** 10.18632/oncotarget.18402

**Published:** 2017-06-07

**Authors:** Tingyin Jiang, Chunling Huang, Yuan Xu, Yingrui Su, Guanjie Zhang, Long Xie, Liqun Huang, Shuchun You, Jinshun Zha

**Affiliations:** ^1^ Department of Nuclear Medicine, Second Affiliated Hospital, Fujian Medicine University, Licheng, Quanzhou 362000, China; ^2^ Cancer Institute, Fudan University Shanghai Cancer Center, Xuhui, Shanghai 200032, China

**Keywords:** thyroid cancer, lymph node ratio, survival, stage IV, medullary

## Abstract

To assess the prognostic value of lymph node ratio (LNR) in patients with stage IV thyroid cancer based on the Surveillance, Epidemiology, and End Results (SEER) database. A total of 4,940 eligible patients were included for the analysis. Kaplan-Meier survival analysis and Cox proportional hazard regression were used to reveal the effect of LNR on overall survival (OS) and disease specific survival (DSS). The optimal cut-off value of LNR for predicting OS and DSS was determined by the time-dependent Receiver Operating Characteristic analysis. By the univariate Cox proportional hazard regression, LNR was significantly associated with OS and DSS in patients with medullary thyroid cancer (MTC), papillary thyroid cancer and anaplastic thyroid cancer (all *P* < 0.05). With the optimal cut-off value, Kaplan-Meier analysis showed that MTC patients with LNR≥76.5% were significantly associated with poorer OS (log-rank test: *P* < 0.0001), and LNR≥40.7% were significantly associated with poorer DSS (log-rank test: *P* < 0.0001). LNR was an independent prognostic factor of poorer survival in MTC patients after adjusting for other variables by multivariable Cox analysis (OS: hazard ratio [HR] = 2.560, 95% confidence interval [CI] 1.690–3.879, *P* < 0.0001; DSS: HR=2.781, 95% CI 1.582–4.888, *P =* 0.0004). Our results demonstrated that LNR could predict clinical outcomes in patients with stage IV MTC, and 76.5% was the optimal cut-off value of LNR to predict OS. LNR, as a function of the nodes positive and the nodes examined, could provide suggestions on the postoperative prognosis of patients with stage IV MTC.

## INTRODUCTION

Thyroid cancer (TC) is the most common endocrine malignancy, and the incidence had been accelerating worldwide over the past few decades [1, 2]. In the United States, it is estimated that about 56,870 cases of TC are expected to be diagnosed and about 2,010 cases of death in 2017 [3]. It is known that first-line preferred treatment is surgical extirpation of tumor, whenever possible, followed by radioactive iodine (RAI) [4]. With the advances in treatment combined with early detection, 5-year survival of TC patients is excellent [5], especially for papillary TC (PTC).

The pathological types of TC, mainly containing papillary, medullary, follicular and undifferentiated, not only differs on pathogenesis, but also on the metastatic behavior and prognosis. For instance, nearly 30% to 80% patients of PTC have regional LN metastasis [6]. But for follicular thyroid cancer (FTC) patients, the most common metastasized region is not LN but distant organs, such as lung and bone. Therefore, the importance of LN dissection is not equivalent to all pathological types of TC patients. Still, therapeutic LN dissection for patients with clinically metastases is performed worldwide [7].

Previous studies have demonstrated the prognostic significance of lymph nodes (LNs) metastases [8, 9], which present the recurrence and progression of TC, is well accepted. However, the significance of LNs metastases on clinical outcomes remains controversial. Historically, some studies reported that disease in LNs could not influence mortality [10]. More recent studies suggested that metastatic LNs negatively influence survival [11, 12]. Therefore, it is of interest to evaluate the prognostic value of LNs on clinical outcomes. Lymph nodes ratio (LNR), estimated by the number of positive LNs to the total number of LNs examined, has been acknowledged as a prognostic factor of survival in gastric, colon, breast, pancreatic, uterine and ovarian cancer [13–18]. However, the clinical benefit of LNR in advanced stage TC is scant. Therefore, we present LNR, as a function of the number of nodes positive and the number of nodes examined, to examine the prognostic value of LNR in stage IV TC patients with different pathological types based on the Surveillance, Epidemiology and End Results (SEER) program.

## RESULTS

### Baseline characteristics and univariate analysis of prognosis

Finally, a total of 4,940 eligible patients with Stage IV thyroid cancer were included in the present study (Table 1), containing four major histological types, PTC (85.2%), FTC (1.1%), MTC (8.4%) and anaplastic TC (ATC, 5.6%). Among the patients, 3717 (75.2%) patients were staged IVA, 482 (9.8%) patients were staged IVB, 554 (11.2%) patients were staged IVC, and 187 (3.8%) patients were staged IVNOS.

**Table 1:** Baseline characteristics and univariate Cox regression analysis of patients with stage IV thyroid cancer

variables	*N* (%)	Overall survival		Disease specific survival	
		HR (95% CI)	*P* value	HR(95%CI)	*P* value
**Age (years)**					
**<45**	125 (2.5)	1.00		1.00	
**≥45**	4815 (97.5)	2.688 (1.485–4.869)	0.001	1.503 (0.804–2.811)	0.202
**Gender**					
**Females**	2620 (53.0)	1.00		1.00	
**Males**	2320 (47.0)	1.466 (1.295–1.661)	< .0001	1.254 (1.056–1.489)	0.010
**Race**					
**White**	4087 (82.7)	1.00		1.00	
**Black**	186 (3.8)	0.979 (0.713–1.345)	0.897	1.453 (0.985–2.142)	0.060
**Asians**	603 (12.2)	0.943 (0.778–1.141)	0.545	0.707 (0.522–0.958)	0.025
**Others**	64 (1.3)	0.651 (0.324–1.306)	0.227	0.728 (0.182–2.918)	0.654
Grade*					
**I**	657 (13.3)	1.00		1.00	
**II**	223 (4.5)	1.636 (1.029–2.601)	0.037	1.784 (0.823–3.866)	0.142
**III**	225 (4.6)	8.591 (6.217–11.870)	< .0001	18.337 (11.045–30.446)	< .0001
**IV**	277 (5.6)	16.307 (12.020–22.122)	<.0001	33.112 (20.247–54.151)	< .0001
**Unknown**	3558 (72.0)	1.768 (1.335–2.342)	<.0001	2.230 (1.382–3.597)	0.001
**AJCC stage**					
**IVA**	3717 (75.2)	1.00		1.00	
**IVB**	482 (9.8)	3.690 (3.111–4.376)	< .0001	5.795 (4.583–7.326)	< .0001
**IVC**	554 (11.2)	6.349 (5.475–7.362)	< .0001	11.107 (9.074–13.596)	< .0001
**IVNOS**	187 (3.8)	3.519 (2.751–4.500)	< .0001	2.662 (1.709–4.147)	< .0001
**Histological types**					
**Papillary**	4029 (85.2)	1.0		1.00	
**Folliculary**	55 (1.1)	2.544 (1.629–3.972)	< .0001	3.991 (2.285–6.972)	< .0001
** Medullary**	416 (8.4)	1.710 (1.386–2.109)	< .0001	2.457 (1.857–3.252)	< .0001
** Anaplastic**	277 (5.6)	11.429 (9.730–13.424)	< .0001	18.636 (15.148–22.928)	< .0001
** Unknown**	163 (3.3)	5.178 (4.091–6.553)	< .0001	7.702 (5.674–10.454)	< .0001
**Radiation treatment**					
** No**	1405 (28.4)	1.0		1.00	
** Radioisotopes**	2927 (59.3)	0.378 (0.327–0.436)	< .0001	0.399 (0.324–0.491)	< .0001
** Beam radiation**	469 (5.5)	1.758 (1.360–2.272)	< .0001	2.979 (2.408–3.686)	< .0001
** Unknown**	139 (2.8)	0.638 (0.379–1.074)	0.091	0.633 (0.324–1.237)	0.181
**Total LNs examined**					
** 1–10**	2063 (41.8)	1.00		1.00	
** 11–20**	859 (17.4)	0.647 (0.536–0.780)	< .0001	0.628 (0.486–0.812)	0.0004
** > 20**	2018 (40.9)	0.728 (0.635–0.835)	< .0001	0.640 (0.528–0.776)	< .0001
**Positive LNs examined**					
** < 5**	2633 (60.0)	1.00		1.00	
** ≥ 5**	2307 (40.0)	0.801 (0.706–0.909)	0.001	0.886 (0.745–1.054)	0.171

Abbreviations: LN-lymph node; *I-well differentiated; II-moderately differentiated; III-poorly differentiatd; IV-undifferentiated; anaplstic.

By the univariate Cox regression analysis, we found that age (≥ 45 years), sex (males), grade, AJCC staging, histological types, radiation treatment, extent of lymphadenectomy and number of positive nodes were all significantly associated with OS (all *P* < 0.05) (Table 1). And gender, race, grade, AJCC staging, histological types, radiation treatment and extent of lymphadenectomy were associated with DSS (all *P* < 0.05). Extent of lymphadenectomy showed a significant association with OS (11–20vs.1–10: hazard ratio [HR] = 0.647, 95% confidence interval [CI] 0.536–0.780, *P* < 0.0001; > 20 vs.1–10: HR = 0.728, 95% CI 0.635–0.835, *P* < 0.0001), and with DSS (11–20 vs. 1–10: HR = 0.628, 95% CI 0.486–0.812, *P* = 0.0004; > 20 vs. 1–10: HR = 0.640, 95% CI 0.528–0.776, *P* < 0.0001). And the number of positive nodes examined had significant effect on OS (≥ 5 vs. <5: HR = 0.801, 95% CI 0.706–0.909, *P* = 0.001), however, the effect was not found on DSS (≥ 5 vs. <5: HR = 0.886, 95% CI 0.745–1.504, *P* = 0.171).

### Prognostic value of LNR on OS stratified by histological types

By the univariate Cox analysis, LNR was examined to be significantly associated with OS in patients with PTC, FTC, MTC and ATC (all *P* < 0.05) (Table 2). After adjusting for age, sex, race, grade, AJCC staging and radiation treatment, multivariate Cox analysis revealed that LNR was an independently prognostic factor of OS in patients with MTC (HR: 3.879, 95% CI 2.004–7.509, *P* < 0.0001).Similarly, LNR was found to increase the risk of disease specific death in patients with PTC, MTC and ATC, even after adjustment for other variables (all *P* < 0.05).

**Table 2:** Univariate and multivariate Cox regression analysis of thyroid cancer patients based on different histological types

Histological types	Univariate analysis		Multivariate analysis	
	HR (95%CI)	*P* value	HR (95% CI)	*P* value
**Overall survival**				
**Papillary**	1.462 (1.150–1.859)	0.002	1.265 (0.856–1.869)	0.238
**Follicular**	3.705 (1.824–7.527)	0.0003	0.511 (0.151–1.729)	0.280
**Medullary**	5.526 (2.901–10.527)	< .0001	3.879 (2.004–7.509)	< .0001
**Anaplastic**	1.637 (1.112–2.410)	0.013	1.626 (1.098–2.406)	0.015
**Disease specific survival**				
**Papillary**	1.910 (1.318–2.767)	0.001	1.491 (1.033–2.152)	0.033
**Follicular**	0.667 (0.165–2.693)	0.569	0.597 (0.140–2.542)	0.485
**Medullary**	5.625 (2.440–11.964)	< .0001	4.174 (1.792–9.722)	0.001
**Anaplastic**	1.614 (1.025–2.540)	0.039	1.597 (1.009–2.528)	0.049

### Identification of appropriate cutoff points of LNR in MTC

**Figure 1:**
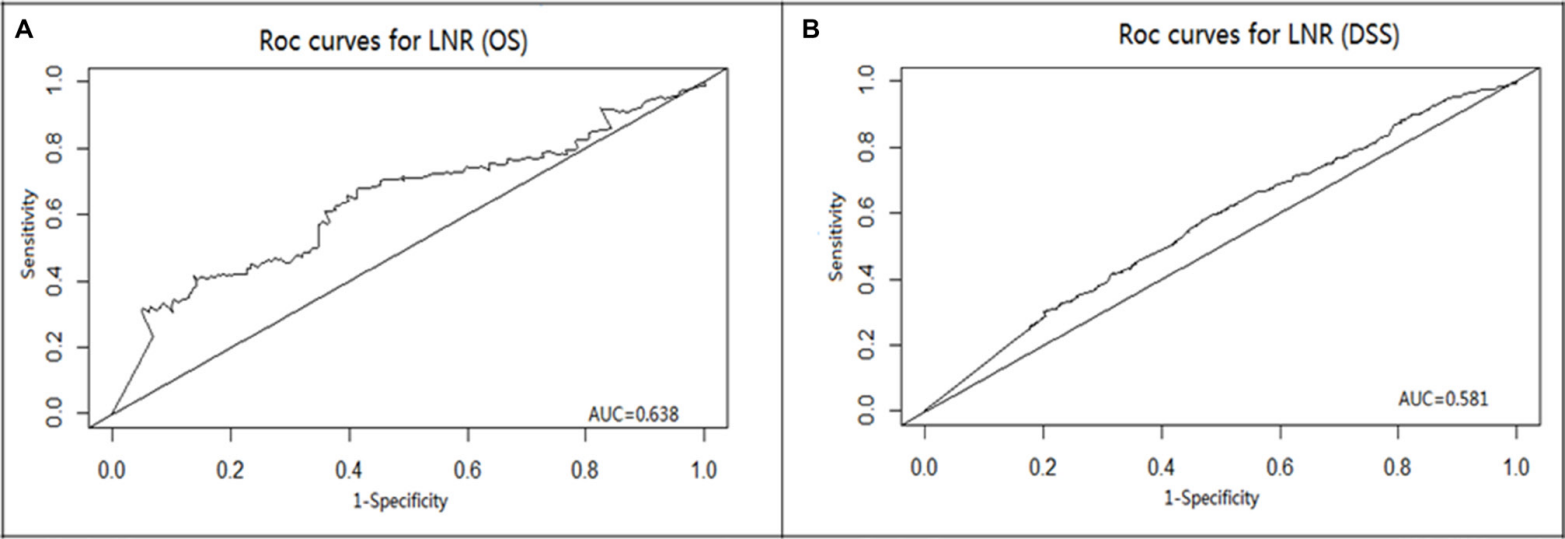
(**A**) Receiver operating characteristic (ROC) curve with LNR in prediction of overall survival in MTC patients with stage IV. (**B**) Receiver operating characteristic (ROC) curve with LNR in prediction of disease specific survival in MTC patients with stage IV.

**Figure 2:**
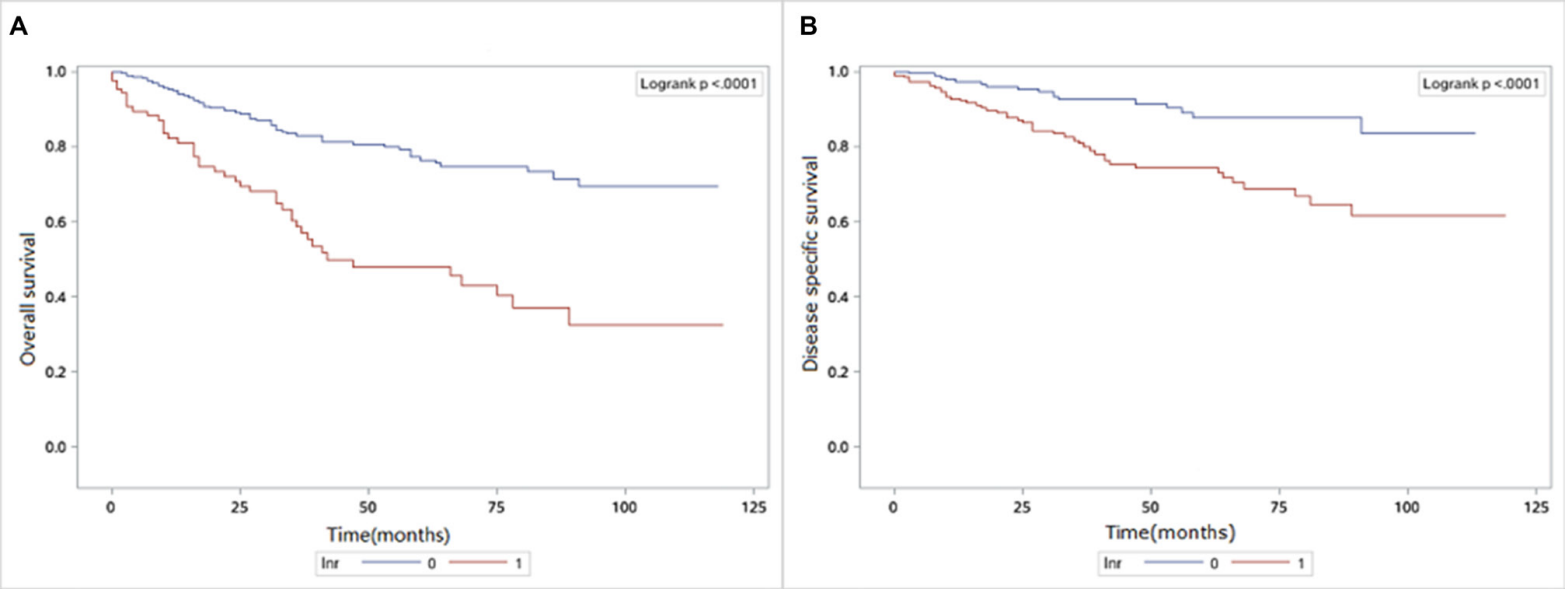
(**A**) Impact of LNR on overall survival in MTC patients with stage IV. (**B**) Impact of LNR on disease specific survival in MTC patients with stage IV.

ROC analysis was used to determine the optimal cut-off value of LNR for prediction of OS and DSS in MTC patients (Figure 1). The results showed that the LNR were 76.5% and 40.7% when the Youden index was the largest, respectively. Therefore, 76.5% and 40.7% were determined as the appropriate cut-off value of LNR for OS and DSS (OS: Area Under Curve = 0.634, *P* < 0.0001; DSS: AUC = 0.581, *P* < 0.0001). Then the MTC patients were divided into two groups by the cut-off values of LNR. The Kaplan-Meier analysis showed that a higher LNR was associated with poorer OS (*P* < 0.0001) (Figure 2A) and DSS ( *P* < 0.0001) (Figure 2B).

Univariate and multivariate Cox analysis were used to identify the significance of LNR on OS and DSS in MTC patients (Table 3).Compared with LNR<76.5%, it showed that LNR≥76.5% was significantly associated with OS (HR=3.091, 95% CI 2.085–4,582, *P* < 0.0001), and could predict OS after adjusting for sex, age, race, grade, AJCC stage and radiation treatment (HR=2.560, 95% CI 1.690–3.879, *P* < 0.0001). As we analyze the predictive value of LNR on DSS, LNR≥40.7% could significantly increase the risk of DSS than LNR<40.7% (HR = 2.927, 95% CI 1.672–5.125,*P* = 0.0002), and remains significant after adjustment for other variables (HR = 2.781, 95% CI 1.582–4.888, *P* = 0.0004).

**Table 3:** Effect of LNR on overall survival and disease specific survival in MTC patients by Cox regression analysis

LNR (%)	Number	Univariate analysis		Multivariate analysis	
		HR (95% CI)	*P* value	HR (95% CI)	*P* value
**Overall survival**					
**LNR<76.5**	314	1.00		1.00	
**LNR≥76.5**	102	3.091 (2.085–4.582)	< 0.0001	2.560 (1.690–3.879)	< 0.0001
**Disease specific survival**					
**LNR<40.7**	212	1.00		1.00	
**LNR≥40.7**	204	2.927 (1.672–5.125)	0.0002	2.781 (1.582–4.888)	0.0004

## DISCUSSION

To the best of our knowledge, this is the first empirically based study on the risk of LNR in patients with advanced stage TC. Current guidelines from the National Comprehensive Cancer Network (NCCN) and American Thyroid Association (ATA) [19] recommend central LN compartment dissection for all patients with clinically involved central nodes and consideration of prophylactic central LN dissection in patients with advanced primary tumors. There is still no consensus regarding the optimal extent of LNs dissection; LNR, as a function of the number of positive nodes and the total number of LNs examined, could not only reduce the potential bias during surgery and pathological examination to a certain extent, but also provide suggestion on lymphadenectomy during the surgery.

Previous studies have identified that LNR was an important prognostic factor in several cancers [8–13]. As for TC, the association between LNR and survival in well-differentiated TC was found to be no significance [20]. Patients with different histological types have different probability of LNs metastasis, thus the patients included in our study were advanced stage with four histological types, different from the previous studies.

PTC is the most common histological type of thyroid cancer and elicits good therapeutic response to surgery and radioiodine treatment. For patients with advanced stage PTC, LNR has been suggested to be useful to predict prognosis by several studies [21, 22]. We confirmed that LNR was associated with DSS in patients with advanced stage by univariate and multivariate Cox regression analysis (*P* < 0.05), illustrating the contribution of LNR to the risk of disease specific death. However, its association with OS could not be confirmed by multivariate Cox regression analysis after adjusting for other variables. Based on the present study, we could not conclude that LNR contributes to morality risk in patients with stage IV PTC. ATC, accounting for 1.3–9.8% of total TC, is the histological type that has the most aggressive progression [23]. Most of ATC patients have an extremely poor prognosis with a median survival between 1.2 and 10 months [24], indicating the importance of prognostic factors. In the present study, we demonstrated that LNR could predict the prognosis of OS and DSS in ATC by univariate Cox regression analysis (*P* < 0.05), and also by Cox multivariate regression analysis (*P* < 0.05) after adjusting for variables. Based on those findings, LNR was also associated with clinical outcomes in patients with advanced stage ATC. Due to the limited number of ATC patients, further analysis was censored.

It is known to us that the rates of nodal metastases were relatively high in patients with MTC. The more LNs removal indicated the more complete excision of tumor during surgery in patients with MTC. According to the latest 2015 ATA guidelines, patients with MTC confined to the neck and cervical LNs should undergo dissection of the central (levels VI–VII) and the involved lateral neck compartment (level II–V). Due to the high rate of occult disease, prophylatic central neck dissection is advised in all cN0 patients [25]. It was reported that metastases in more than 10 LNs and involvement of more than two LNs compartments were strongly associated with survival in MTC patients [26]. The value of LNs dissection could be quantified by LNR, which is able to reflect the quality of surgery. Previous study have demonstrated that LNR could predict cancer-specific survival in MTC patients (HR 2.622, 95% CI 1.327–3.519, *P* = 0.002) [27], which was consistent with our results (HR = 4.174, 95% CI 1.792–9,722, *P* = 0.001). The difference of our study is the patients included that they were staged as advanced stage. And by the cutoff value of LNR for MTC patients, it was demonstrating that 76.5% LNR was a potential value to distinguish patients from higher risk of overall death, and 40.7% LNR could also predict the risk of disease specific death. Although the AUC=0.569 was not fully enough to explain the contribution of LNR on the mortality risk of disease specific death, LNR could still predict OS and provide more clinical significance for the postoperative treatment in advanced stage MTC patients.

Although the study reveals numerous important positive findings, it has several potential drawbacks. The main limitation of the study is the inherent bias, which exists in retrospective study. Patients included were from SEER database, thus some cases without complete information was deleted. Also, both the LNs dissected by surgeons and LNs examined by the pathologists could influence the outcome of the study. Second, the SEER database does not provide information on treatment, which could not be adjusted during the multivariate regression analysis. In addition, the study was based on the stage IV MTC patients with limited number of patients, further study with larger samples is needed to validate our results.

In conclusion, we investigated the prognostic value of LNR in patients with stage IV TC based on SEER database. Our results indicated that a higher LNR was significantly associated with poorer OS and DSS in MTC patients with advanced stage who experience surgical treatment with node positive, and 76.5% LNR was the optimal cut-off point to predict OS in MTC patients. LNR, as a function of the nodes positive and the nodes examined, could provide evaluation of lymphadenectomy and give suggestions on the prognosis of postoperative MTC patients.

## MATERIALS AND METHODS

### Patients

Data was obtained from the SEER program, which consists of 18 population-based cancer registries (for more details: https://seer.cancer.gov/registries/terms.html). The registries could broadly represent the Unite States population as a whole, accounting for 28% of population from academic and nonacademic hospitals. In view of the data from SEER were de-identified, written informed consent cannot be assessed. The present study was conducted in accordance with the Declaration of Helsinki and relevant guidelines, and was approved by the independent ethics committee/institutional review board at the Second Affiliated Hospital of Fujian Medical University.

Patients underwent surgical staging followed by lymphadenectomy were identified from SEER database between 1973 and 2013. All of the patients were diagnosed as stage IV thyroid cancer with the excluded criteria as followings: 1) with unknown age records, 2) with benign or borderline tumors, 3) without or with unknown positive nodes, 3) without or with unknown number of node examined. Pathologic diagnosis was based on the primary site by the International Classification of Disease for Oncology, Third Edition (ICD-O-3). Totally, patients’ information on age, sex, race, AJCC staging, grade, histological types, radiation treatment, total number of LNs examined, positive number of LNs examined (LNs containing metastases), overall survival (OS) and disease specific survival (DSS) were extracted into the analysis.

### Statistical analysis

Cox proportional hazard regression analysis was used to identify risk factors of OS and DSS. In the subgroups of histological types, univariated and multivariated Cox regression analysis was used to identify the risk of LNR on OS and DSS. The optimal cut-off value for LNR in medullary TC (MTC) was determined by the time-dependent receiver operating characteristic (ROC) analysis. Then the comparisons between groups divided by the optimal cut-off point of LNR was determined by the Kaplan-Meier method. Finally, univariated and multivariated Cox regression analysis was used to reveal the effect of LNR on survival in MTC with adjusting for age, sex, race, AJCC staging, grade and radiation treatment. All *P-*value < 0.05 was considered as statistically significant. SAS (version 9.4, SAS Institute, Cary, NC) program was used for analysis.
